# Supplementary material for: Modeling glioblastoma heterogeneity as a dynamic network of cell states
Source: Mol Syst Biol. 2021 Sep 16;17(9):e10105. doi: 10.15252/msb.202010105 (PMC8444284; doi:10.15252/msb.202010105)
Supplement: Supplementary file 6 — Source Data for Figure 5 [file MSB-17-e10105-s004.zip › Figure5A_sourcedata/GSEA_3017/hallmarks_stateF.GseaPreranked.1621934628066/HALLMARK_MYC_TARGETS_V1.html]

Details for gene set HALLMARK\_MYC\_TARGETS\_V1[GSEA]

|  || Dataset | state33017 |
| Phenotype | NoPhenotypeAvailable |
| Upregulated in class | na\_neg |
| GeneSet | HALLMARK\_MYC\_TARGETS\_V1 |
| Enrichment Score (ES) | -0.6650668 |
| Normalized Enrichment Score (NES) | -4.157978 |
| Nominal p-value | 0.0 |
| FDR q-value | 0.0 |
| FWER p-Value | 0.0 |
Table: GSEA Results Summary

  

Fig 1: Enrichment plot: HALLMARK\_MYC\_TARGETS\_V1      
 Profile of the Running ES Score & Positions of GeneSet Members on the Rank Ordered List

  

| PROBE | GENE SYMBOL | GENE\_TITLE | RANK IN GENE LIST | RANK METRIC SCORE | RUNNING ES | CORE ENRICHMENT || 1 | RPS10 |  |  | 119 | 0.717 | -0.0377 | No |
| 2 | UBE2E1 |  |  | 1050 | 0.300 | -0.4601 | No |
| 3 | TARDBP |  |  | 1480 | -0.252 | -0.6522 | No |
| 4 | SNRPG |  |  | 1486 | -0.254 | -0.6484 | No |
| 5 | CCT2 |  |  | 1494 | -0.258 | -0.6455 | No |
| 6 | HNRNPA2B1 |  |  | 1503 | -0.261 | -0.6429 | No |
| 7 | PSMD1 |  |  | 1532 | -0.267 | -0.6494 | No |
| 8 | NOLC1 |  |  | 1542 | -0.270 | -0.6471 | No |
| 9 | NPM1 |  |  | 1543 | -0.270 | -0.6406 | No |
| 10 | RNPS1 |  |  | 1548 | -0.271 | -0.6359 | No |
| 11 | LSM7 |  |  | 1551 | -0.272 | -0.6303 | No |
| 12 | SNRPD2 |  |  | 1596 | -0.285 | -0.6438 | No |
| 13 | RUVBL2 |  |  | 1599 | -0.286 | -0.6379 | No |
| 14 | SNRPA |  |  | 1637 | -0.296 | -0.6479 | No |
| 15 | GSPT1 |  |  | 1653 | -0.303 | -0.6475 | No |
| 16 | CTPS1 |  |  | 1692 | -0.316 | -0.6575 | Yes |
| 17 | TRIM28 |  |  | 1709 | -0.322 | -0.6572 | Yes |
| 18 | GNL3 |  |  | 1716 | -0.324 | -0.6522 | Yes |
| 19 | PABPC4 |  |  | 1728 | -0.329 | -0.6493 | Yes |
| 20 | EIF3B |  |  | 1734 | -0.332 | -0.6437 | Yes |
| 21 | HSPD1 |  |  | 1742 | -0.336 | -0.6388 | Yes |
| 22 | GLO1 |  |  | 1755 | -0.342 | -0.6362 | Yes |
| 23 | RPS2 |  |  | 1770 | -0.348 | -0.6343 | Yes |
| 24 | PPM1G |  |  | 1771 | -0.348 | -0.6259 | Yes |
| 25 | RAN |  |  | 1772 | -0.350 | -0.6175 | Yes |
| 26 | HNRNPA3 |  |  | 1776 | -0.351 | -0.6105 | Yes |
| 27 | DDX18 |  |  | 1785 | -0.355 | -0.6056 | Yes |
| 28 | SRM |  |  | 1786 | -0.355 | -0.5971 | Yes |
| 29 | SRPK1 |  |  | 1788 | -0.356 | -0.5890 | Yes |
| 30 | CCT3 |  |  | 1791 | -0.358 | -0.5814 | Yes |
| 31 | RSL1D1 |  |  | 1793 | -0.359 | -0.5732 | Yes |
| 32 | ILF2 |  |  | 1795 | -0.360 | -0.5650 | Yes |
| 33 | HNRNPC |  |  | 1804 | -0.362 | -0.5600 | Yes |
| 34 | SMARCC1 |  |  | 1810 | -0.369 | -0.5535 | Yes |
| 35 | EIF4A1 |  |  | 1822 | -0.376 | -0.5495 | Yes |
| 36 | XRCC6 |  |  | 1825 | -0.377 | -0.5414 | Yes |
| 37 | C1QBP |  |  | 1828 | -0.379 | -0.5332 | Yes |
| 38 | LDHA |  |  | 1833 | -0.380 | -0.5259 | Yes |
| 39 | CDC45 |  |  | 1836 | -0.382 | -0.5177 | Yes |
| 40 | CCT4 |  |  | 1840 | -0.384 | -0.5098 | Yes |
| 41 | FBL |  |  | 1845 | -0.386 | -0.5024 | Yes |
| 42 | PSMA7 |  |  | 1856 | -0.393 | -0.4976 | Yes |
| 43 | POLD2 |  |  | 1868 | -0.398 | -0.4931 | Yes |
| 44 | TCP1 |  |  | 1875 | -0.401 | -0.4863 | Yes |
| 45 | SYNCRIP |  |  | 1877 | -0.403 | -0.4771 | Yes |
| 46 | SRSF1 |  |  | 1879 | -0.405 | -0.4678 | Yes |
| 47 | RFC4 |  |  | 1887 | -0.413 | -0.4611 | Yes |
| 48 | HDAC2 |  |  | 1896 | -0.418 | -0.4548 | Yes |
| 49 | POLE3 |  |  | 1901 | -0.426 | -0.4464 | Yes |
| 50 | ABCE1 |  |  | 1902 | -0.426 | -0.4362 | Yes |
| 51 | SERBP1 |  |  | 1938 | -0.447 | -0.4416 | Yes |
| 52 | PSMA4 |  |  | 1946 | -0.451 | -0.4340 | Yes |
| 53 | NAP1L1 |  |  | 1951 | -0.455 | -0.4250 | Yes |
| 54 | NOP56 |  |  | 1953 | -0.456 | -0.4145 | Yes |
| 55 | EIF1AX |  |  | 1959 | -0.458 | -0.4058 | Yes |
| 56 | HSP90AB1 |  |  | 1963 | -0.461 | -0.3961 | Yes |
| 57 | PTGES3 |  |  | 1970 | -0.467 | -0.3876 | Yes |
| 58 | EIF3J |  |  | 1976 | -0.471 | -0.3786 | Yes |
| 59 | NHP2 |  |  | 1982 | -0.476 | -0.3695 | Yes |
| 60 | MCM4 |  |  | 1993 | -0.487 | -0.3625 | Yes |
| 61 | TRA2B |  |  | 1998 | -0.490 | -0.3525 | Yes |
| 62 | XPO1 |  |  | 2007 | -0.500 | -0.3442 | Yes |
| 63 | RRM1 |  |  | 2023 | -0.516 | -0.3388 | Yes |
| 64 | RANBP1 |  |  | 2024 | -0.517 | -0.3264 | Yes |
| 65 | LSM2 |  |  | 2035 | -0.524 | -0.3184 | Yes |
| 66 | SNRPA1 |  |  | 2040 | -0.528 | -0.3076 | Yes |
| 67 | CCT5 |  |  | 2042 | -0.529 | -0.2953 | Yes |
| 68 | SRSF7 |  |  | 2056 | -0.544 | -0.2883 | Yes |
| 69 | BUB3 |  |  | 2078 | -0.596 | -0.2837 | Yes |
| 70 | SRSF3 |  |  | 2081 | -0.606 | -0.2700 | Yes |
| 71 | HNRNPR |  |  | 2083 | -0.612 | -0.2558 | Yes |
| 72 | USP1 |  |  | 2084 | -0.612 | -0.2411 | Yes |
| 73 | HNRNPU |  |  | 2090 | -0.628 | -0.2283 | Yes |
| 74 | SET |  |  | 2095 | -0.639 | -0.2148 | Yes |
| 75 | SNRPD1 |  |  | 2105 | -0.653 | -0.2033 | Yes |
| 76 | DDX21 |  |  | 2107 | -0.659 | -0.1879 | Yes |
| 77 | KPNB1 |  |  | 2108 | -0.660 | -0.1721 | Yes |
| 78 | CDC20 |  |  | 2117 | -0.695 | -0.1591 | Yes |
| 79 | SRSF2 |  |  | 2122 | -0.706 | -0.1440 | Yes |
| 80 | PA2G4 |  |  | 2123 | -0.707 | -0.1270 | Yes |
| 81 | HDGF |  |  | 2124 | -0.709 | -0.1100 | Yes |
| 82 | HNRNPD |  |  | 2126 | -0.713 | -0.0933 | Yes |
| 83 | ODC1 |  |  | 2128 | -0.718 | -0.0766 | Yes |
| 84 | DEK |  |  | 2154 | -0.817 | -0.0685 | Yes |
| 85 | DUT |  |  | 2155 | -0.824 | -0.0487 | Yes |
| 86 | MCM7 |  |  | 2160 | -0.839 | -0.0304 | Yes |
| 87 | KPNA2 |  |  | 2178 | -0.894 | -0.0168 | Yes |
| 88 | CCNA2 |  |  | 2181 | -0.902 | 0.0039 | Yes |
| 89 | MAD2L1 |  |  | 2228 | -1.204 | 0.0115 | Yes |
Table: GSEA details [plain text format]

  

Fig 2: HALLMARK\_MYC\_TARGETS\_V1: Random ES distribution      
 Gene set null distribution of ES for **HALLMARK\_MYC\_TARGETS\_V1**
